# Prof. Coventry’s Report on Cholera

**Published:** 1848-08

**Authors:** 


*Prof. Coventry's Report on Cholera.*—In January last, we published some resolutions adopted by the Medical Faculties of the Geneva and Buffalo Medical Colleges, commissioning Prof. Coventry, to obtain the latest information in Europe, as to the pathology and treatment of Asiatic Cholera, which, as it was then reported, had made its appearance in France and Great Britain. The latter intelligence, fortunately, proved to be erroneous, and Prof. C., in consequence, did not, witness the disease. The subject, however, was under earnest discussion in France and England, owing to the expectancy of the speedy appearance of the disease there, and Prof. C. was able to obtain the views of some of those who had had the largest opportunities for its investigation, and to compare them with his own views derived from a close observation of the epidemic as it appeared in this country in 1832. Prof. C. has embodied the results of his inquiries and experience in a report which we have the satisfaction to present in this No. of the Journal. In view of the possibility, if not probability, of the reappearance of this terrible scourge upon our shores, we commend the report to the careful perusal of our readers.

